# A Prospective Study on the Association between Oxidative Stress and Duration of Symptoms in Allergic Rhinitis

**DOI:** 10.3390/jpm11121290

**Published:** 2021-12-03

**Authors:** Hyun Moon, Changsun Sim, Jiho Lee, Inbo Oh, Taehoon An, Jongcheol Lee

**Affiliations:** 1 Department of Otorhinolaryngology, Gangneung Asan Hospital, University of Ulsan College of Medicine, Gangneung 25440, Korea; m3k1mh@gmail.com (H.M.); ebeenooo@gmail.com (T.A.); 2 Department of Occupational and Environmental Medicine, Ulsan University Hospital, University of Ulsan College of Medicine, Ulsan 44033, Korea; drsimcs@gmail.com (C.S.); leejh@uuh.ulsan.kr (J.L.); 3 Environmental Health Center, Ulsan University Hospital, University of Ulsan College of Medicine, Ulsan 44033, Korea; oinbo@naver.com

**Keywords:** allergic rhinitis, total oxidant status, total antioxidant status, prospective study, school children

## Abstract

Oxidative stress has been known to play an important role in inflammatory responses of allergic rhinitis. We investigated the association between degree of oxidative stress and severity of allergic rhinitis. A total 226 allergic rhinitis students were classified by a history of allergic rhinitis into groups according to number and duration of symptoms within the previous year. The total antioxidant status (TAS) and total oxidant status (TOS) levels were compared among groups. Mean TAS level (14.03 ± 9.09 mmol/L) in the group with more than six months of symptoms had a tendency to be higher (*p* = 0.068) than that of the group with fewer than six months (12.33 ± 8.83 mmol/L). There was no statistically significant difference in mean TAS or TOS level with number of symptoms (nasal congestion, itching, sneezing and watery rhinorrhea). A multivariate logistic regression showed that the adjusted odds ratio of TAS was 1.655 and the adjusted odds ratio of TOS was 0.972 in more than a six-month duration group. The TAS level was significantly associated with a more than six-month symptom duration (*p* = 0.034). Our results suggest that antioxidant activity increased when allergic rhinitis became chronic and further research will be needed considering the disease severity.

## 1. Introduction

The concept of oxidative stress, which was introduced in 1985 for research in redox biology and medicine, refers to the imbalance between oxidants and antioxidants in the body. Oxidation is any chemical reaction that moves electrons from one substance to another. The oxidizing agent is chemically reduced by removing electrons from its reaction partners, which thus are oxidized. Antioxidants are chemically-reduced compounds that slow or prevent the oxidation process. As a result of this chemical reaction, antioxidants can protect other compounds from being oxidized. Oxidants are substances that produce and increase reactive oxygen species (ROS) and reactive nitrogen species in the body. Excessive amounts of ROS cause imbalance between oxidative forces and antioxidant defense systems, leading to oxidative injuries that deform and damage the structure of surrounding substances and resulting in loss of function. Oxidative stress has been posited to play an important role in human aging and various diseases [1,2,3].

Among the various organs of the human body, the respiratory system is exposed to the highest degree of oxidative stress. Oxidative stress has been known to play a major role in the pathogenesis, clinical course, and treatment of asthma. Oxidants and other reactive oxygen metabolites are increased in various biological samples of patients with asthma, and the level of antioxidants in the body is decreased. Additionally, increased environmental oxidative stress such as air pollution is known to be associated with increased incidence of asthma, worsening of its symptoms and severity, and decreased pulmonary function [4,5,6].

In allergic rhinitis (AR), which has a similar epidemiologic and immunopathological background as asthma, many reports have demonstrated that oxidative stress and its resultant production of ROS play an important role in its inflammatory responses. Excessive ROS can enhance mucosal permeability and increase mucus secretion accompanied with an influx of inflammatory cells and can induce injury to respiratory epithelial cell layers by decreasing the number and functionality of epithelial cilia. Moreover, ROS activate transcription factors in inflammatory cells to produce messengers, which lead to expression of genes for many pro-inflammatory cytokines, enzymes, and adhesion molecules [7]. However, studies on the effect of oxidative stress on AR are limited, with most targeting only a few individual oxidant and antioxidant markers without considering the overall oxidant or antioxidant status of the patients [8,9,10,11]. Our previous study, which investigated the association between overall systemic oxidative stress and AR, indicated that children with AR had higher serum total oxidant status (TOS) and total antioxidant status (TAS) than children without AR. Children with AR also had an elevated oxidative stress index (OSI), which is an objective value of oxidative stress conditions. In other words, patients with AR have systemically elevated oxidative stress and systemically elevated TAS level [12].

In our current study, which is an extension of our previous study, we aim to investigate the association between degree of oxidative stress and severity of AR. An underlying association between severity of AR and degree of oxidative stress could be a scientific basis for the role of oxidative stress in the pathogenesis and treatment of AR. Because the effects of individual antioxidants are additive, and measurement of all individual antioxidants is almost impossible, we measured serum TAS and TOS, which reflect overall systemic antioxidation and oxidation states, respectively. After classifying children with a history of AR into groups according to number and duration of symptoms within the previous year, we compared and analyzed the TAS and TOS levels among groups.

## 2. Materials and Methods

### 2.1. Study Participants

This study was conducted as a retrospective analysis of a prospective database in a single center and was approved by the local institutional review board. A large, school-based cohort allergy study was performed in 2010 by the Ulsan University Hospital Environmental Health Center in Republic of Korea. The study participants were recruited from an elementary school in an area with the same local environment, responded to a questionnaire, and underwent routine medical checkups, including a skin prick test and a blood test. A questionnaire was given to the participant’s parents or legal guardians. The Korean version of the International Study of Asthma and Allergies in Childhood questionnaire was used [13,14]. The parents or legal guardians of all participants provided written informed consent. Upon completion of the questionnaire, participant blood serum and urine samples were collected and frozen for further study.

Of the 1403 students in the elementary school, 1387 responded to the questionnaire, 1322 (95.3%) completed the questionnaire, and 571 (41.2%) underwent medical checkups. The presence of AR was defined if a child’s parents reported “yes” to the question, “Has your child been treated for AR by a doctor in his or her lifetime?”. Among children who had been treated with AR by a doctor, the question, “Has your child ever had one or more symptoms of nasal congestion, itching, sneezing, or watery rhinorrhea, even though he or she did not have a cold or flu over the last 12 months?”, was used to determine the symptoms for the previous year. In addition, the question, “In the last 12 months, how long were your child’s symptoms present?”, was used to determine the duration of symptoms in persons who had symptoms during the previous year. Of the 571 students who underwent medical checkups, 303 had been treated for AR by a doctor, and 263 showed symptoms during the last year. This study included 226 students who had symptoms during the previous year, underwent medical checkups, and completed the questionnaire.

### 2.2. TAS and TOS Measurements

The serum samples used for measurement of TAS and TOS were stored at −80 °C until analysis with a commercially available assay kit (Rel Assay Diagnostics; Mega Tip, Gaziantep, Turkey), developed by Erel [15,16]. The automated method for TAS measurements is based on bleaching of the characteristic color of a more stable ABTS (2,2′-azino-bis[3-ethylbenzothiazoline-6-sulfonic acid]) radical cation by antioxidants. Antioxidants in the sample reduce dark blue colored ABTS radicals to a colorless reduced ABTS form. The change in absorbance at 660 nm is related to TAS of the sample. The assay is calibrated with a stable antioxidant standard solution, which is traditionally called Trolox equivalent and is a vitamin E analog. The results are expressed as millimoles of Trolox equivalent per liter.

Color intensity, which can be measured spectrophotometrically for TOS measurements, is related to the total amount of oxidant molecules in the sample. That is, oxidants present in the sample oxidize the ferrous ion-chelator complex to ferric ion. This oxidation reaction is enhanced by abundant glycerol molecules in the reaction medium. The ferric ion forms a colored complex with xylenol orange in an acidic medium. The assay is calibrated with hydrogen peroxide, and the results are expressed in micromoles of hydrogen peroxide equivalent per liter.

### 2.3. Statistical Analysis

Children who had symptoms during the previous year were classified according to number of symptoms and duration. We used *t*-test for comparison of TAS/TOS among group classified according to symptom duration. ANOVA was used to compare TAS/TOS levels according to the number of symptoms. We analyzed the associations between the duration of symptoms of chronic allergic rhinitis and TOS/TAS level after adjusting the other variables using multivariate logistic regression. Statistical analyses were performed with SPSS 21 software (IBM Corp., Armonk, NY, USA) and Excel 2010 (Microsoft Corp., Redmond, WA, USA). A value of *p* < 0.05 was considered significant.

## 3. Results

### 3.1. Characteristics of the Study Participants

A total of 226 children who had symptoms during the previous year were included in this analysis. The mean (SD) age was 8.96 (1.75) years, the mean (SD) height was 137.21 (11.59) cm, and the mean (SD) weight was 35.45 (10.23) kg. Skin prick tests for inhalation allergens showed 127 (56.2%) positive cases, all of whom were positive for more than one allergen (Table 1). There were 143 (63.3%) children who had been diagnosed by a doctor with atopic disease apart from AR (i.e., asthma, allergic conjunctivitis, and allergic dermatitis) during the previous year, and 83 (16.8%) children had never been diagnosed with atopic disease.

### 3.2. Distribution of Number and Duration of Symptoms

Of the 226 children who had symptoms during the previous year, 155 (68.6%) had nasal congestion, 87 (38.5%) had nasal itching, 60 (26.5%) had watery rhinorrhea, and 59 (26.1%) had sneezing. With regard to symptom number, 138 (61.1%) had only one symptom, 57 (25.2%) had two symptoms, 15 (6.6%) had three symptoms, and 16 (7.1%) had four symptoms. The distribution of symptom duration showed 35 (15.5%) children with symptoms lasting less than overall one month in a year. Symptoms lasted more than two months in 191 cases (84.5%), more than four months in 92 cases (40.7%), more than six months in 42 cases (18.6%), and throughout the 12 months in 18 cases (8.0%). According to the monthly symptom distribution, the most frequent month was December, in which 158 children (69.9%) showed symptoms, and the least frequent month was July, in which 20 (8.8%) showed symptoms. The distribution of seasonal symptoms shows that 142 children (62.8%) had symptoms in spring, 32 (14.2%) in summer, 160 (70.8%) in autumn, and 174 (77.0%) in winter.

### 3.3. Comparison of TAS and TOS

The overall mean (SD) TAS level was 1.40 (0.73) mmol/L (range: 1.26–1.67), and the overall mean (SD) TOS level was 13.72 (9.05) mmol/L (range: 0.50–33.45). To examine the association between symptom duration of AR and degree of oxidative stress, the mean TAS and TOS levels were compared according to symptom duration. The mean (SD) TAS and TOS levels in the group with less than one month of symptoms were 1.41 (0.07) mmol/L and 15.23 (9.79) mmol/L, respectively, compared to those of the group with more than one month of symptoms, which were 1.40 (0.07) mmol/L and 13.45 (8.90) mmol/L. There was no significant difference in mean TAS or TOS level. In groups classified according to the two-month duration, there was no significant difference in mean TAS or TOS level. However, in groups classified according to a six-month duration, mean TAS level (14.03 ± 9.09 mmol/L) in the group with more than six months of symptoms had a tendency to be higher than that of the group with fewer than six months of symptoms (12.33 ± 8.83 mmol/L) with no statistically significant difference (*p* = 0.068) (Table 2).

**Figure 1:**
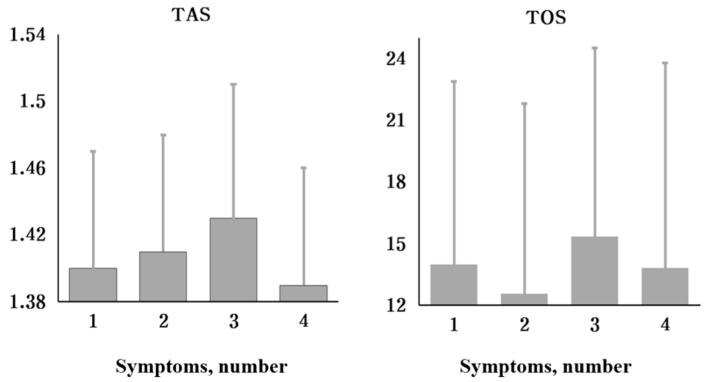
Mean total antioxidant status (TAS) total oxidant status (TOS) among groups classified according to number of symptoms.

We divided the subjects into groups I, II, III, and IV according to number of symptoms and compared mean TAS/TOS levels of the groups by ANOVA test (Figure 1). There was no statistically significant difference in mean TAS or TOS level with increasing number of symptoms (Table 3). In the group with more than six months of symptoms, the relationships between mean TAS and TOS levels and increasing number of symptoms were not statistically significant (Table 4).

### 3.4. Associations between Symptom Duration and Mean TAS and TOS Levels

A multivariate logistic regression was conducted with other clinical variables to examine the associations between symptom duration and mean TAS and TOS levels. In the group with more than six-month symptom duration, the adjusted odds ratio (aOR) of TAS was 1.655 (95% CI, 1.040–2.634), and the aOR of TOS was 0.972 (95% CI, 0.935–1.011) compared to that of the group with less than a six-month duration. The TAS level was significantly associated with the group with a more than six-month symptom duration (*p* = 0.034) (Table 5).

## 4. Discussion

In this study, we found that TAS and TOS levels did not show significant difference in the groups classified according to number of symptoms. The TOS level was not associated with groups classified according to duration of symptoms, but the TAS level was significantly higher in the group of children with more than 6 months of symptoms, as confirmed by multivariate logistic regression analysis. In our previous report, we found that both serum TAS and TOS levels were significantly higher in the AR group compared to a control group. In other words, serum oxidative stress was increased in allergic rhinitis patients; however, there was no difference in serum oxidative stress depending on number of symptoms. Additionally, antioxidant activity increased when AR became chronic. This finding might be regarded as an adaptive response to the chronic oxidative stress. This may also imply the possibility that antioxidants may be helpful in chronic AR. However, several clinical trials of antioxidants as an additional therapeutic for asthma and chronic obstructive pulmonary disease (COPD) have not demonstrated any efficacy. Suggested reasons are that antioxidants cannot travel to exactly where they are needed, and that the dose of antioxidants used in clinical trials is limited so blood antioxidant concentrations cannot be sufficiently increased [5].

We arbitrarily classified the study population according to number and duration of symptoms experienced during the previous one year. The Allergic Rhinitis and its Impact on Asthma (ARIA) classification considered duration of symptoms as one of the important factors for health-related quality of life and reported that this is an important factor in deciding treatment agents of AR [17]. In other studies, the visual analogue scale (VAS) of symptoms and the total four-symptom score, which was calculated as the sum of the four typical nasal symptoms of AR (nasal itching, rhinorrhea, nasal congestion, and sneezing), were used to evaluate severity of AR [18,19]. We have also conducted study using the number of symptoms according to previous studies. However, since the number of symptoms does not mean the severity of the allergic rhinitis, it is thought that a validated scale should be used in future studies to find out the relationship between disease severity of allergic rhinitis and oxidative stress.

The most important strength of the current study was that the study participants were recruited from a single elementary school in an area with the same local environment. The difference of local environments can play an important role in the occurrence and progression of AR, and this can be a difficult interfering factor in observational studies. Many studies have reported that exposure to air pollution and high-concentration micro-dust was associated with increased risk of asthma, AR, and allergic sensitization. Previous studies also found that meteorological and environmental factors were closely correlated with AR incidence [20,21,22]. Enrollment of students from a single elementary school in an area with the same local environment can reduce interference of environmental factors on the results of the study.

There are some limitations of this study. First, the use of surveys has been studied, and shows that even with a standardized questionnaire, the sensitivity of identification of atopic sensitization to airborne allergens is modest at best. The results of surveys on AR relying solely on questionnaires must therefore be considered with caution [23]. Moreover, since our study had no control group, it is possible that the effects of other diseases or environments that cause oxidative stress, such as parental smoking, were not considered. The other limitation of this study was that we did not evaluate the subjects’ daily diets and did not investigate the type and duration of any treatment. Tamay et al. reported that some pro-Mediterranean foods (such as cereals, rice, pasta) and chocolates may have a beneficial effect on symptoms of AR, while other foods such as animal fats and candies may have aggravating effects [24]. Saadeh et al. reported that frequent consumption of a healthy diet including meat, fish, and fruits seems to have a protective effect on prevalence of asthma-related symptoms and allergic sensitization [25]. The results of these studies demonstrate that dietary habits may affect the prevalence of AR symptoms. Another important point is that the treatment of AR could affect the results of this study. Antihistamines had been reported to increase total antioxidant status, and steroids were reported to reduce oxidative stress in an animal model of asthma and inhibit oxidative phosphorylation and ROS generation [26,27,28]. Further study is required to determine the role of food and medication in systemic TAS/TOS of AR. Furthermore, we only used the number of symptoms to evaluate the severity of allergic rhinitis. In addition, a large number of participants were recruited until the questionnaire stage, but it is regrettable that many of them did not respond to medical check-up and the number of participants decreased significantly. Finally, it would have been helpful in interpreting our results if additional inflammatory cytokine assay as well as TAS/TOS was conducted. However, despite these shortcomings, this study was meaningful as it elucidates the role of TOS/TAS in AR and provides a basis for guidelines on use of antioxidant therapy. We think it adds significant clinical information to this field.

## 5. Conclusions

The TAS level was high when the symptom duration was more than 6 months, and the TOS level was not. Our results suggest that antioxidant activity increased when AR became chronic. Further research will be needed considering not only the symptom duration but also the disease severity.

## Figures and Tables

**Table 1:** General characteristics of the study population.

Variables		Case (N: 226)
Age (yr), mean ± SD		8.96 ± 1.75
Sex, male:female (%)		136:90 (60.2%:39.8%)
Height (cm), mean ± SD		137.21 ± 11.59
Weight (kg), mean ± SD		35.45 ± 10.23
Grade (%)	1st	34 (15.0%)
	2nd	27 (11.9%)
	3rd	33 (14.6%)
	4th	50 (22.1%)
	5th	33 (14.6%)
	6th	49 (21.7%)
Skin prick test (%)	Positive	127 (56.2%)
	Negative	99 (43.8%)

**Table 2:** Comparison of TAS/TOS among groups classified according to duration of symptoms.

Symptom Duration	N	TAS		TOS	
		Mean ± SD	*p* Value	Mean ± SD	*p* Value
<1 month	35	1.41 ± 0.07	0.717	15.23 ± 9.79	0.283
≥1 month	191	1.40 ± 0.07		13.45 ± 8.90	
<2 months	88	1.40 ± 0.08	0.653	14.61 ± 9.01	0.240
≥2 months	138	1.41 ± 0.07		13.16 ± 9.06	
<3 months	134	1.40 ± 0.07	0.486	14.00 ± 9.01	0.577
≥3 months	92	1.41 ± 0.07		13.31 ± 9.13	
<6 months	184	1.40 ± 0.07	0.068	14.03 ± 9.09	0.271
≥6 months	42	1.42 ± 0.08		12.33 ± 8.83	

N, number; SD, standard deviation; TAS, total antioxidant status; TOS, total oxidant status.

**Table 3:** TAS and TOS (mean ± SD) among groups classified according to number of symptoms.

Variables	I	II	III	IV	*p* Value *
TAS (mmol/L) Mean ± SD	1.40 ± 0.07	1.41 ± 0.07	1.43 ± 0.08	1.39 ± 0.07	0.299
TOS (µmol/L) Mean ± SD	14.00 ± 8.90	12.57 ± 9.23	15.36 ± 9.18	13.84 ± 9.94	0.674

I, group of patients with one symptom of allergic rhinitis; II/III/IV, group of patients with two/three/four symptoms; SD, standard deviation; TAS, total antioxidant status; TOS, total oxidant status. * Calculated by ANOVA *t*-test.

**Table 4:** Comparison of TAS/TOS (mean ± SD) among groups classified according to number and six-month duration of symptoms.

Symptom Duration		Groups According to Number of Symptoms				*p* Value
		I	II	III	IV	
<6 months	N	127	41	8	8	
	TAS	1.40 ± 0.07	1.41 ± 0.07	1.39 ± 0.04	1.38 ± 0.07	0.741
	TOS	13.97 ± 8.97	13.87 ± 9.55	15.12 ± 10.31	14.98 ± 9.00	0.975
≥6 months	N	11	16	7	8	
	TAS	1.40 ± 0.08	1.42 ± 0.08	1.48 ± 0.08	1.40 ± 0.06	0.154
	TOS	14.45 ± 8.46	9.25 ± 7.65	15.64 ± 8.51	12.71 ± 11.30	0.320

I, group of patients with one symptom of allergic rhinitis; II/III/IV, group of patients with two/three/four symptoms; N, number; TAS, total antioxidant status; TOS, total oxidant status.

**Table 5:** Summary of multiple logistic regression of TAS/TOS levels in the group with symptom duration more than six months.

Variable	Category	Number	*p* Value	Exp(B)	95% CI	
					Lower	Upper
Skin prick test	Negative	99	0.056	2.052	0.982	4.288
	Positive	127				
Age	<10 years.	131	0.366	0.715	0.346	1.479
	≥10 years.	95				
Sex	Boys	136	0.217	0.625	0.296	1.318
	Girls	90				
AR history of father	No	158	0.816	1.094	0.513	2.332
	Yes	68				
AR history of mother	No	142	0.887	1.053	0.515	2.155
	Yes	84				
TAS		226	0.034	1.655	1.040	2.634
TOS		226	0.163	0.972	0.935	1.011

AR, allergic rhinitis; CI, confidence interval; TAS, total antioxidant status; TOS, total oxidant status.

## Data Availability

The data presented in this study are available on request from the corresponding author. The data are not publicly available due to privacy.
